# The costs of the economic crisis in the health sector

**Published:** 2009

**Authors:** Paul Radu, Victor Purcărea, Florian Popa

**Affiliations:** * Medical University Carol Davila Bucharest, Romania; ** National School of Public Health and Health Services Management, Bucharest, Romania

## Abstract

**Introduction**:

The Romanian health care system is facing a period of deep transformations, having in the last years some clear achievements, but also some acknowledged problems. Once the world’s economic crisis appeared and manifested its first effects in Romanian economy, there will be some consequences in the health sector too. Regarding the Romanian social health insurance scheme, it’s possible to assist to greater financial constraints in 2009 due to reduced contributions and a weaker economy. There are two possible solutions to face this challenge: diminishing the basic health services covered by the social health insurance system (difficult to be accepted by politicians and population) and better use of available resources based on an improvement of allowance and technical efficiency.

**Materials and methods**:

The authors have made a critical analysis of the Romanian health care system facing the forecasted effects of the economic crisis and have proposed some interventions and solutions to reduce the effects at the level of the patient. In order to increase allowance efficiency clear measures are necessary to be taken at central and local level: improvement of financing mechanisms, alignment of the incentives used by the payment mechanisms for different type of providers, development of guides and protocols based on available resources, etc. For increased technical efficiency there is a need for adequate incentives within the financing and organization mechanisms in order to make the public providers more active in obtaining greater outputs from the available resources. The economic constraints will lead to the growth of the quality of medical services. This is the reason why the health system needs the development of the accreditation systems for providers. The audit of medical activities and reporting represents a mechanism which needs to be developed. Regarding the new health technologies’ use new institutions and mechanisms need to be implemented. They will allow making certain decisions on health technologies’ use based on cost-effectiveness criteria. The efficient use of the available resources cannot be done without the proper incentives system for professionals who provide health care services. It means that we should establish a better relationship between the professional individual performance and the official revenues.

**Results**:

It’s clear that the actual economic crisis will induce effects in the health care sector. It is up to the Government and the physicians, professional bodies and patient’s associations to reach a consensus regarding the introduction and the development of some appropriate rules and mechanisms, which will have less effects of the crisis at the patient’s level.

## Introduction

Within the European Union (EU) the health sector is seen as one of the main priorities for all citizens and, consequently it should rise to population’s expectations in terms of: protection against diseases and illnesses, access to support and high quality health care services (the article 152 of EU Treaty specifies that “A high level of human health protection shall be ensured in the definition and implementation of all Community policies and activities”).

The development of a health care system is directly related with its financing and the amount (%) of Gross Domestic Product (GDP) assigned to the health sector; the results being observed in a low rate of morbidity and mortality, early stage diagnosis of diseases and the improvement of cost-effectiveness services. Moreover, the funding of the health sector represents the generator of health reforms, due to poor coverage of the necessary expenditure in health sector, based on increasingly higher demands. 

The Romanian health care system is facing a period of deep transformations. Some of the achievements are clear: free and equal access to public health services, involvement and empowerment of public administration in the development and implementation of health care programs, decisional transparency in funds allocation, simplification of financial flows regarding money transfer from central to local administration, the capacity of building and planning regulations of the Ministry of Health on objectives, activities and structures of public health sector. But, there are also some problems, such as the late implementation of the integrated health management information system created for a better use of resources and available funds.

Once the world’s economic crisis appeared and manifested its first effects in Romanian economy we have to be ready to accept and fight its consequences in the health care system. The main determinants for limitations within the health care sector will be: fewer financial resources at the economy level, reductions of capital investment, increased costs of credits, etc.

## Materials and methods

The Romanian social health insurance scheme functions at this moment based on a shared percentage of the contribution system, employer (5,5%) and employees (5,2%) with a total of 10,7% from revenues. This 10,7% contribution represents a much smaller share than the one insurance turned out, in 1999, when it was 14%. Meantime, it’s possible to assist in 2009, not only at this reduced contribution, but also at a reduced number of contributors as a consequence of a weaker economy. Thus, it’s easy to forecast that there will be greater financial constraints in 2009 within the social health insurance scheme, especially if we also bare in mind the constant pressure for increased salaries in the health sector.

There are two possible solutions to face this challenge induced by the economic crisis in the social health insurance system:

1. Diminishing the basic health services covered by the social health insurance system. This drastic action is difficult to be accepted by the new Parliament and Government, due to a highly emotional impact on the population and also taking into consideration that new Presidential Elections will take place in 2009. The international experience in this area shows that it’s very difficult to cut out the existing basic health services covered by the health insurance, by using cost-effectiveness criteria (because the politicians and population are expecting an increase of the covered services, not a decrease!). For example, in 2007 a referendum took place in Hungary in order to establish if to introduce some co-payments for the hospital care or not, which was rejected by the population. In Romania, in 2008 (election year), an enlargement of the compensated drug list took place and new health services covered by the public health insurance schemes (treatment of obesity, in-vitro fertilization and HPV vaccine for girls aged 11 years old) were introduced.

2. Better use of available resources. From this perspective new health policies, health instruments, financing tools, etc., are expected to be introduced for an improvement of allowance and technical efficiency.

Allowance efficiency means the use of limited resources in directions that produce a maximum welfare, meaning a maximization of the impact on the health status. For example, there are several illnesses that can be treated in ambulatory settings, day-hospitalization or continuous hospitalization. The costs associated with these alternative settings are very different, but frequently the outcomes are identical. Despite this, there is a very heterogeneous medical practice and few protocols to be used by the clinicians in order to provide care with fewer costs. Moreover, the actual payment mechanisms do not give incentives to provide care in the settings with lower costs. It’s obvious that, if the providers get only 1/3 of the usual tariff for a patient treated in a day-hospitalization compared to the same condition treated in continuous hospitalization care, they will not use the day-hospitalization, because they prefer increased revenues with continuous hospitalization, even if the costs associated are higher.

The conclusion is simple: in order to increase allowance efficiency several clear, urgent and fair measures at central and local level are necessary: improvement of financing mechanisms, alignment of the incentives used by the payment mechanisms for different types of providers, simple regulations regarding the patients’ flow within the social health insurance system, development of guides and protocols based on available resources, etc.

Technical efficiency covers the way the resources are used at the provider level in order to produce maximum outputs. The fewer resources are used to get the results, the better the efficiency. But, how the Romanian providers are stimulated to be efficient nowadays? The answer is clear: apart from the private providers who want an increased profit, the rest of the providers (most of them) have no incentives to be efficient. This is very obvious at the hospitals’ level, which spend a lot of resources without incentives to be technically efficient, due to the fear of historical payments that rewards the inefficient settings, fear of reducing structures or personnel. And the hospital stakeholders, meaning the actual health administration, have never asked “profit” from hospitals, knowing that among central rules personnel norms, salaries, etc are imposed. In this context, it worth to study the way the actual pilot project at the level of Bucharest (the transfer of 18 hospitals under the subordination of Bucharest Mayoralty) will bring not only some financial support from the Mayoralty, but also the incentives for the hospitals to be more efficient.

The economic constraints will impose an increase of the quality of medical services, because the lack of quality induces services’ duplication and increased costs. The lack of quality is seen quite often at the level of some health services and providers, where the confidence regarding the results of some health activities done by some professionals is questioned by their peers. This shows that people from inside the health system recognize a lack of quality, even if, from the perspective of technical and material resources, progress has been made during the last years. This is the reason why the health system needs a development of the accreditation systems for providers, that could bring with it better achievement of the quality standards and a diminished dissipation of resources through duplication of investigations, analysis, etc.

The audit of medical activities and reporting represents a mechanism which needs to be developed in order to avoid services duplication, to pay the providers according to their activities and to respect the principle that “money follows the patient”. To support the audit mechanism we need not only a declaration in the legal documents, but also personnel and resources to make it credible and reliable. We need to descend with the support of auditing mechanism from the central payer level towards providers, professional associations and patient associations. The experiences from other countries show that simple measures like peers revue, double-check of medical record, web availability of data regarding performance and results etc., could bring improvements in provider’s activity and reduced expenditures.

Regarding the new health technologies use, more of them expensive, but with better efficacy, we need the implementation of new institutions and mechanisms which will allow making certain decisions on health technologies use at central level, based on cost-effectiveness criteria, instead of decisions taken by individuals at the provider level. The experience of institutions from other countries (like NICE - National Institute of Health and Clinical Excellence from Great Britain) could be very useful in the development of local mechanisms which take into consideration cost-effectiveness criteria. 

The efficient use of available resources could not be done without the proper incentives system for professionals that provide health care services. It means that we should establish a better relationship between the professional individual performance and the official revenues. Meantime, if we know that from a financial perspective “money follows the patient“ from an organizational perspective “the patient follows the physician”. It means that if we want to provide more ambulatory services (with less costs), we need to develop mechanisms to attract physicians out of hospitals, because the patients will get out of hospitals together with the physicians. But, as long as the hospital represents the principal “attraction” for physicians (from all perspectives: professional, academic and financial), it will be very difficult to introduce effective mechanisms to move patients towards ambulatory care.

## Results

It’s clear that the actual economic crisis will induce effects in the health care sector. It is up to the Government together with physicians, professional bodies and patient associations to reach a consensus regarding the introduction and development of the appropriate rules and mechanisms in order to have less effects of the crisis at the patient’s level.

These things could be done with courage and ability to invent and act with perseverance so that the economic crisis is used only as the catalyst for a deep reform of the health care system in patient’s benefit. And, as we described, the more the changes in the health sector, the better the dynamics and the dimension of the process change will be related, not only with political willingness and subjective hopes, but also with the presence and sufficiency of objective necessary conditions.
